# Temporal trend and characteristics of notifications of self-inflicted and interpersonal violence in the transgender population in Brazilian municipalities, 2015-2022

**DOI:** 10.1590/S2237-96222024v33e2024296.especial.en

**Published:** 2024-12-06

**Authors:** Kelly Roberta Estrela Marinho, Jeane Tomazelli, Vania Reis Girianelli

**Affiliations:** 1 Fundação Oswaldo Cruz, Programa de Pós-graduação em Saúde Pública, Rio de Janeiro, RJ, Brazil; 2 Instituto Nacional de Câncer, Divisão de Ensino Stricto Sensu, Rio de Janeiro, RJ, Brazil; 3 Fundação Oswaldo Cruz, Departamento de Direitos Humanos, Saúde e Diversidade Cultural, Rio de Janeiro, RJ, Brazil

**Keywords:** Personas Transgénero, Violencia de Género, Sistema de Información en Salud, Derechos Humanos, Epidemiología Descriptiva., Transgender People, Gender-Based Violence, Health Information System, Human Rights, Descriptive Epidemiology

## Abstract

**Objective:**

To analyze the temporal trend and characteristics of notifications of violence among the transgender population from 2015 to 2022 in Brazilian municipalities.

**Methods:**

This was a repeated panel epidemiological study, based on violence incidents reported among the transgender population aged 20 to 59 years, available in the Notifiable Health Conditions Information System. An annual temporal trend analysis was performed by means of generalized linear regression, using the Prais-Winsten method and spatial distribution of notifying municipalities in Brazil.

**Results:**

Notifications of violence in the transgender population decreased during the period (1.7%; β = -0.07; p = 0.010), but there was an increase in the number of notifying municipalities (45.8%), self-inflicted violence (28.9%; β = 2.21; p < 0.001) and sexual violence (β = 0.79; p < 0.001). The majority of perpetrators were male and in an affective relationship, especially with transgender women (43.4%; p < 0.001).

**Conclusion:**

Notification of violence does not yet fully reflect the reality of this population, but it represents the first step towards visibility and addressing the issue.

## Study contributions

**Main results:**

Notifications of violence against transgender people accounted for 1.7% of the total. Self-inflicted and sexual violence increased from 2015 to 2022. Almost half of Brazilian municipalities have already been reporting cases of violence against this population.

**Implications for services:**

Continuous and high-quality notification will contribute to monitoring and understand violence in this population group, enabling the adaptation of services to meet their specific needs.

**Perspectives:**

The development of research on the transgender population will allow for a better understand and guidance of specific health actions for this group. Information on violence against this population is crucial for informing public policies.

## RESUMEN

**Objetivo:**

Analizar tendencias temporales y características de notificaciones de violencias ocurridas entre la población transgénero de 2015 a 2022, en municipios brasileños

**Métodos:**

Estudio epidemiológico de panel repetido, basado en datos de violencia ocurridos entre población transgénero de 20-59 años, disponibles en el Sistema de Información de Enfermedades de Declaración Obligatoria. Análisis de tendencia temporal anual, se realizó mediante regresión lineal generalizada, utilizando método de Prais-Winsten y distribución espacial de municipios brasileños notificados.

**Resultados:**

Las notificaciones de violencia entre la población trans disminuyeron en el período (1,7%; β = -0,07; p = 0,010), pero con un aumento en municípios reportados (45,8%), violencia autoinfligida (28,9%; β = 2,21; p < 0,001) y sexual (β = 0,79; p < 0,001). La mayoría de los agresores son hombres y mantienen una relación sentimental, especialmente en caso de mujeres transgénero (43,4%; p < 0,001).

**Conclusión:**

Denunciar la violencia aún no refleja la realidad de esta población, pero es el primer paso hacia su visibilización y enfrentamiento.

## INTRODUCTION

Violence is a complex concept whose damage to physical and psychological health impacts not only health and safety, but also other areas of society, such as education and the economy. Therefore, it must be prioritized in public policies developed in the country.^1^ Since 1893, death due to violence has been included in the International Classification of Diseases.^2^ However, it was not until the 1990s that the Pan American Health Organization, in response to the increasing morbidity and mortality from violence in Latin America, recommended the inclusion of violence in the intervention agenda.^3^ Subsequently, the World Health Organization recognized violence as a preventable public health problem issue, and organized the first global report containing recommendations for its management.^1^

Violence is characterized by the intentional use of force or power that may cause injury, death, deprivation, disability or psychological harm.^1^ Self-inflicted violence refers to acts of self-harm, ranging from minor injuries to severe wounds and even suicidal behavior, while interpersonal violence involves the use of physical force, power or psychological influence to dominate or exclude another person. Interpersonal violence is subdivided into domestic and community violence. As for domestic violence, perpetrators are family members, intimate partners or individuals who share the domestic space, even occasionally, such as employees and relatives, and this type of violence can occur outside the family environment. Regarding community interpersonal violence, the perpetrators are either strangers or acquaintances, but without family or emotional ties.^4^

In 2001, Brazil established the National Policy for the Reduction of Morbidity and Mortality from Accidents and Violence,^5^ and in 2004, the National Network for Violence Prevention and Health Promotion was implmented.^6^ Since 2011, notification of self-inflicted and interpersonal violence has been part of the compulsory notifications list^7^ and has been included in the Notifiable Health Conditions Information System (*Sistema de Informação de Agravos de Notificação* - SINAN), allowing for records from across country, although the legislation had already incorporated notification of violence against children and adolescents, the elderly, women and suicide. In 2015, to align with the National Policy for Comprehensive Health of Lesbians, Gays, Bisexuals, Transvestites and Transgender (*Política Nacional de Saúde Integral de Lésbicas, Gays, Bissexuais, Travestis e Transexuais*),^8^ the variables “sexual orientation” and “gender identity” were included in the notification form.^4^

Gender identity is the way an individual perceives and expresses themselves socially, and when it differs from their biological sex, the individual is referred to as transgender.^9^ It is estimated that 0.7% of the Brazilian adult population is transgender.^10^ This population is among the most vulnerable to violence, due to the great individual and institutional discrimination they experience.^11^ Brazil has the highest number of transgender people murdered, especially transvestites and transgender women, reflecting a profound intolerance toward diversity.^12^ ^,13^

Notification of interpersonal and self-inflicted violence among the transgender population enables the monitoring of this population health problem and informs management about necessary intervention actions to reduce and prevent it.^14^ This study aims to analyze the temporal trend and characteristics of notifications of violence among the transgender population between 2015 and 2022, in Brazilian municipalities.

## METHOD

This was a repeated panel epidemiological study, a hybrid study combining a cross-sectional and follow-up study,^15^ on self-inflicted and interpersonal violence in the transgender population aged 20 to 59 years, registered in SINAN, in the period from 2015 to 2022, with preliminary data for 2021 and 2022. The files related to the compressed anonymized microdata were downloaded from the website of the Brazilian National Health System Information Technology Department (*Departamento de Informática do Sistema Único de Saúde*), in the first half of December 2023. 

The annual temporal trend for the period was described using the year of notification as the independent variable and the proportion of violence against transgender individuals in relation to the total number of notifications of the age group studied, as well as the proportion of the type of violence (self-inflicted, interpersonal and unknown) among transgender individuals and the proportion of each type of interpersonal violence (physical, psychological, etc.) as the dependent variable. The temporal trend analysis was performed by means of generalized linear regression, using the Prais-Winsten method. In cases where the trend showed statistical significance (p ≤ 0.05), the Durbin -Watson statistic (d) was used, considering an eight-year series, where results between 1.332 and 2.668 confirm that there is no residual autocorrelation.^16^

The spatial distribution of Brazilian municipalities with and without notifications of violence (self-inflicted and interpersonal) against the transgender population aged 20 to 59 years was presented in a choropleth map, using the TabWin program version 4.1.5, for the initial year of the study series (2015), before the pandemic (2019), during the pandemic (2020) and at the end of the pandemic (2022). The proportion of municipalities in each state and region with notification was also calculated.

Furthermore, the available variables were analyzed: gender, socioeconomic characteristics (age group, race/skin color, and schooling), same municipality of residence and notification (yes; no), characteristics of the perpetrator (number, sex, and relationship), and motivation for the aggression. The percentage of each category of variables was calculated, stratifying the socioeconomic and demographic characteristics by type of notification (self-inflicted and interpersonal) and the characteristics of interpersonal violence by gender (transgender man and transgender woman, including transvestites). Pearson’s chi -square test was used to assess the existence of a statistically significant difference between the strata (p ≤ 0.05), with Yates’ correction, if necessary.

The data were analyzed using the R statistical software version 4.2.1 by means of the foreign, MASS, prais and read.dbc packages.

## RESULTS

**Figure 1:**
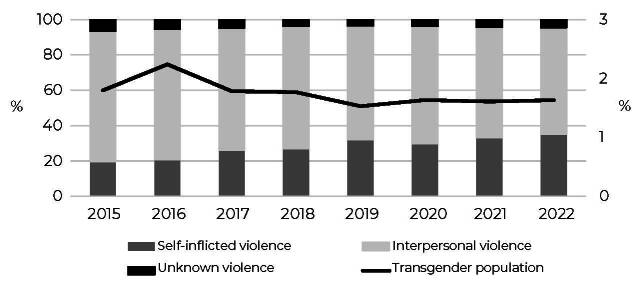
Percentage distribution of notifications of violence in the transgender population, aged between 25 and 59 years, and by type of notification according to year, Brazil, 2015 to 2022

A total of 2,778,017 cases of violence were reported during the study period, with 1,535,329 occurring in the 25-59 age group (55.3%). Of these, 26,258 (1.7%) were related to the transgender population, showing a downward trend during the period (β = -0.07; p = 0.010; d = 1.71) and with the highest percentage of notification in 2016 (2.2%) (Figure 1). Among the notifications in the transgender population, the majority were related to interpersonal violence (66.6%), although this showed a decrease over the period (β = -1.94; p < 0.001; d = 2.21). However, the percentage of notifications of self-inflicted violence (28.9%), increased (β = 2.21; p < 0.001; d = 1.88), representing 35% of the notifications in 2022. On the other hand, the percentage of notifications classified as unknown regarding the type of violence remained stable (4.5%; p = 0.157).

**Figure 2:**
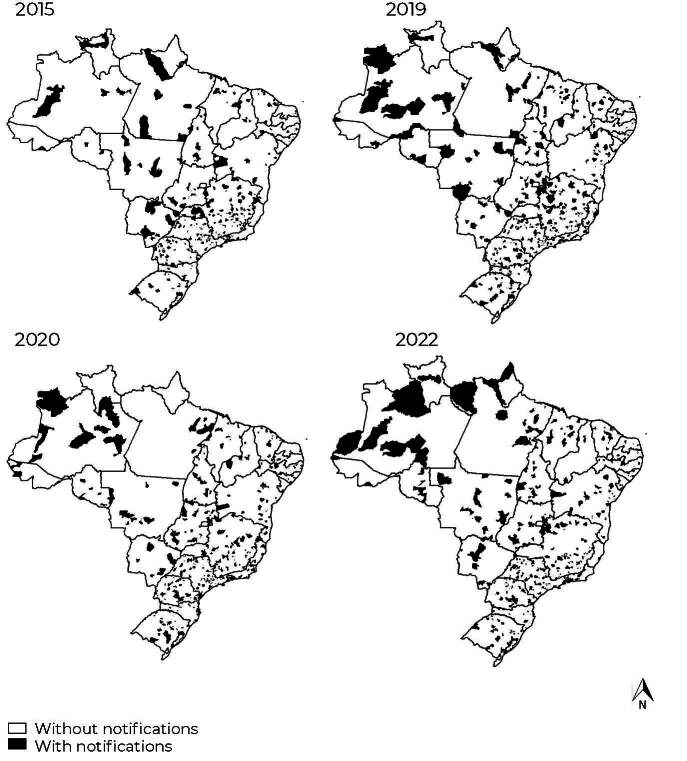
Municipalities with notifications of self-inflicted and interpersonal violence among the transgender population aged 25 to 59, Brazil, 2015, 2019 (before the pandemic), 2020 (during the pandemic) and 2022

Figure 2 shows the maps of Brazilian municipalities with notifications of violence against the transgender population in the age group studied. In 2015, 13,2% of municipalities reported violence, which increased to 18.2% in 2019 (before the pandemic), then dropped to 16.4% in 2020 (during the pandemic) and rose to 19.0% in 2022. In the period 2015 to 2022, 45.8% of municipalities reported violence against the transgender population. The Southeast region showed the highest percentage of municipalities with notifications (61.3%), with the highest proportion in the state of Rio de Janeiro (70.7%). The Northeast region had the lowest percentage of notifying municipalities (37.8%), but with a high percentage in the state of Rio Grande do Norte (79.2%), and a low percentage in the states of Sergipe (24.0%), Piauí (15.2%) and Paraíba (12.6%). Over half of the municipalities in the North region reported violence, with a high percentage in the state of Roraima (80.0%).

The notification of self-inflicted violence was proportionally higher among transgender men (22.4%; p < 0.001), those under 40 years of age (78.6%; p < 0.001) and of White race/skin color (45.9%; p < 0.001) compared to interpersonal violence (Table 1). Schooling showed a high percentage of unknown information, which was proportionally higher for notification of self-inflicted violence (31.9%; p < 0.001). Approximately 90% of notifications were made in the municipality of residence, for both self-inflicted and interpersonal violence (p = 0.094).

**Table 1:** Percentage distribution of sociodemographic characteristics of the transgender population, according to type of violence, Brazil, 2015 to 2022

**Characteristics**	**Type of violence**				**p-value**
	**Interpersonal**		**Self-inflicted**		
	**n**	**%**	**n**	**%**	
**Gender**					
Transgender man	3.142	18.0	1.702	22.4	< 0.001
Transgender woman	14,342	82.0	5,881	77.6	
**Age range**					
20 to 39	13.112	75.0	5,962	78.6	
40 to 59	4,372	25.0	1.621	21.4	< 0.001
**Race/skin color**					
White	6.188	35.4	3,481	45.9	< 0.001
Black	10.032	57.4	3,638	48.0	
Asian	144	0.8	58	0.8	
Indigenous	205	1,2	69	0.9	
Unknown	915	5.2	337	4.4	
**Schooling**					
Illiterate	162	0.9	57	0.8	< 0.001
Incomplete elementary school	3,975	22.7	1,394	18.4	
Complete elementary school	1.305	7.5	449	5.9	
Incomplete or complete high school	5,658	32.4	2,592	34.2	
Incomplete or complete higher education	1,350	7.7	674	8.9	
Unknown	5.034	28.8	2,417	31.9	
**Same municipality of residence and notification**					
Yes	15,807	90.4	6.803	89.7	0.094
No	1,677	9.6	780	10.3	
**Total**	17,484	100.0	7,583	100.0	-

**Figure 3:**
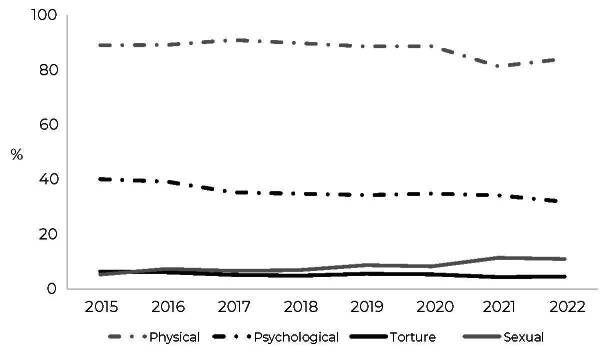
Percentage distribution of the nature of notification of interpersonal violence in the transgender population aged 20 to 59, by year, Brazil, 2015 to 2022

Multiple types of interpersonal violence were reported in 32.5% of notifications. The majority were cases of physical violence (87.5%), followed by psychological violence (35.3%), but both decreased over the period (β = -0.10; p = 0.041; d = 1.87 and β = -1.06; p = 0.003; d = 1.64, respectively) (Figure 3). Torture also showed a decrease over the period (β = -0.25; p = 0.011; d = 1.91), with the highest percentage of notification in 2015 (6.5%) and the lowest in 2021 (4.4%). Conversely, sexual violence increased (β = 0.79; p < 0.001; d = 2.04), accounting 8.5% of notifications during the period, reaching 11.5% in 2021. Notifications of sexual violence were proportionally higher for transgender men during the period (9.2%; p < 0.001) (data not shown in tables).

Most notifications of interpersonal violence involved a single perpetrator (69.0%), and was committed by males (80.7%), with cases involving more than one perpetrator and those involving perpetrators of both sexes were proportionally higher for transgender men (34.4%; p < 0.001 and 3.7%; p = 0.026, respectively) (Table 2). Regarding the relationship between the perpetrator and the victim, 41.0% involved an affective relationship – spouse, ex-spouse, boyfriend/girlfriend, ex-boyfriend/girlfriend –, being proportionally higher for transgender women (43.4%; p < 0.001); followed by strangers (20.6%), with a higher proportion for transgender men (24.7%; p < 0.001). Violence perpetrated by acquaintances and family members was also proportionally higher for transgender men (21.6% and 12.5%, respectively; p < 0.001). The majority of motives for violence was ignored (32.7%), with a higher proportion for transgender women (33.5%). 

**Table 2:** Percentage distribution of notifications of interpersonal violence in the transgender population, by characteristics of the perpetrators and motivation, Brazil, 2015 to 2022

**Characteristics**	**Total**		**Transgender** **man**		**Transgender** **woman**		**p-value**
	**n**	**%**	**n**	**%**	**n**	**%**	
**Number of perpetrators**							
One	11,409	69.0	1.909	60.8	9,500	66.2	< 0.001
Two or more	5.133	31.0	1,080	34.4	4.053	28.3	
Unknown	942	5.4	153	4.9	789	5.5	
**Sex of the perpetrator(s)**							
Masculine	13,347	80.7	2,417	76.9	10,930	76.2	0.026
Feminine	2.629	15.9	466	14.8	2163	15.1	
Both	566	3.4	117	3.7	449	3.1	
Unknown	942	5.4	140	4.5	802	5.6	
**Perpetrator’s bonds**							
Family members^a^	1,821	10.4	393	12.5	1,428	10.0	< 0.001
Affective relationship^b^	7.174	41.0	945	30.1	6.229	43.4	
Caregiver	27	0.2	7	0.2	20	0.1	
Acquaintance(s)	2,943	16.8	680	21.6	2.263	15.8	
Stranger(s)	3,595	20.6	777	24.7	2.818	19.6	
Institutional^c^	446	2.6	91	2.9	355	2.5	
Others^d^	369	2.2	76	2.4	293	2.0	
Unknown	1,327	7.5	389	12.4	938	6.5	
**Motive for violence**							
Sexism	2.008	11.5	246	7.8	1,762	12.3	< 0.001
Transphobia	1,578	9.0	426	13.6	1,152	8.0	
Generational conflict	2,565	14.7	492	15.7	2.073	14.5	
Homelessness	946	5.4	246	7.8	700	4.9	
Deficiency	128	0.7	17	0.5	111	0.8	
Others	4,547	26.0	813	25.9	3,734	26.0	
Unknown	5.712	32.7	902	28.7	4.810	33.5	
**Total**	17,484	100.0	3.142	100.0	14,342	100.0	-

a) Father, mother, stepfather, son, brother; b) Spouse, ex-spouse, boyfriend, ex-boyfriend; c) Boss/boss, institutional relationship, police officer/law enforcement agent; d) Includes racism, religious intolerance and xenophobia, 0.1% of each.

## DISCUSSION

The study found that 1.7% of the notifications of self-inflicted and interpersonal violence were recorded among the transgender population, reaching 2.2% in 2016. A survey conducted between November and December 2018 estimated that the transgender population aged 18 years and older in the country was 0.7%,^10^ with a lower proportion only in the Midwest region (0.2%). However, it is noteworthy that the transgender population is among the most vulnerable to intolerance and discrimination; therefore, a higher proportion of notifications of violence compared to other groups is expected. In addition, the survey^10^ was conducted during a period of rising far-right political influence in the country, characterized by conservative moral discourse and strong criticism of “gender ideology,”^17^ which may have discouraged some participants from responding. However, the percentage of notifying municipalities increased during the period, reaching 19.0% in 2022, but almost half of the municipalities had already reported in the period. Furthermore, approximately 90% of notifications occurred in the municipality of residence, indicating engagement from health services. A study on notifications of interpersonal violence against women aged 20 to 59 years, conducted between 2015 and 2021, found that 31.7% of Brazilian municipalities had recorded cases involving transgender people.^18^

The proportion of notifications of self-inflicted violence has increased, and was proportionally higher among transgender men, of White race/skin color who were young compared to interpersonal violence. Similar results were found in a study based on medical records from a transgender outpatient clinic in the Federal District,^19^ where suicidal ideation was more frequent among transgender men (80.5%), aged 18 to 40 years, and with incomplete high school or higher education (60.6%). A study conducted among transgender adults living in the states of São Paulo and Rio Grande do Sul found that 67.7% had suicidal ideation and 43.1% had already attempted suicide.^20^ A study conducted with transgender women living in São Paulo identified a history of sexual violence as a factor associated with suicidal ideation and attempts, even after adjusting for other statistically significant factors.^21^

The majority of notifications of violence in the transgender population concerned women, with 82.0% of cases of interpersonal violence and 77.6% of cases of self-inflicted violence. A study conducted between 2016 and 2020, using data from TabNet on interpersonal violence in the city of São Paulo, also found a higher percentage of notifications among transgender women.^22^

The proportion of notifications of interpersonal violence among the transgender population decreased over the period, but there was an increase in sexual violence, which was higher among men. However notifications of sexual violence have been higher for cisgender women than for transgender women.^18^ Corrective rape, intended to force the individual to change their sexuality to conform to heteronormativity,^23^ is a form of violence that has been reported among cisgender lesbian women, but transgender men also susceptible to this type of violence. Corrective and collective rape now carry aggravated penalties under the Penal Code.^24^

Physical violence remains the most reported type of violence, but it decreased from 2019 to 2021, which may be related to the difficulty in accessing health services due to the COVID-19 epidemic. The classification of offensive, prejudiced and violence acts against LGBTQIAPN+ people as equivalent to the crime of racism in 2019,^25^ may also have influenced the reduction in notifications. The decrease in notifications does not necessarily mean a reduction in violent practices among the transgender population, whose resurgence of physical violence in 2022 nearly returned to the levels seen at the beginning of the series (2015). A study that analyzed violence against women found a higher proportion of notifications of physical violence against transgender women .^18^

Psychological violence was the second most frequent type of violence, indicating its recognition by both victims and professionals. However, unlike physical violence, it continued to decline in 2022. Psychological violence usually emerges at the beginning of the cycle of aggression, especially in intimate relationships, and therefore tends to be less visible. It is more frequently revealed in interview-based studies,^26^ physical and sexual violence in women victims of intimate partner violence assisted in the primary care services. METHODS This is a cross-sectional study, conducted in 26 health units in Vitória, State of Espírito Santo, from March to September 2014. We interviewed 991 women aged 20-59 years. To classify the psychological, physical and sexual violence, the World Health Organization instrument on violence against women was used and a questionnaire to investigate the sociodemographic, behavioral characteristics, and the women’s family and life history was developed. The statistical analyzes used were Poisson regression, Fisher’s exact test and Chi-square. RESULTS The prevalence we observed were psychological 25.3% (95%CI 22.6–28.2and can often only be identified by the interviewer.

Most perpetrators were biologically male (80.7%) and had an intimate relationship with the victim (41.0%), with a higher proportion of cases involving transgender women (p < 0.001). A study focused on violence against women found that aggression from people with an emotional bond to the victim was higher among transgender women.^18^ A systematic review and meta-analysis on intimate partner violence in the transgender population^27^CINAHL found a lifetime prevalence of 37.5% for this type of violence, with transgender people being more likely to experience intimate partner violence compared to the cisgender population. This study included 74 articles from various countries published up to 2019, two of which were Brazilian, although the main focus was on HIV.

The motive for the violence was not recorded in 32.7% of the notifications, making it impossible to identify the main causes of aggression. The notification of violence perpetrated due to gender identity (transphobia), sometimes assumed by the aggressor in relation to the victim, is crucial for informing public policies aimed at combating this discrimination. Schooling also showed a high percentage of missing data, which has been observed in studies based on information systems.

The low completion of some fields in the notification form is one of the limitations of the study, as well as the potential for data entry errors, given that some concepts may still be unfamiliar to health professionals, as evidenced by some studies.^28^ ^,29^ The lack of information on gender identity in the demographic census also hinders the estimation of the prevalence of violence in the transgender population, which is essential information for supporting public policies. Existing estimates, despite efforts at representativity, include a small sample size and tend to focus on large urban centers. 

Additionally, the likely underreporting is expected to be higher in this type of harm, as it depends on recognition by both the victim and the health professional, a situation that is not always perceived by both. Partnerships with non-governmental organizations, including notification, as provided by law, could help minimize existing underreporting, improve the quality of information and better direct services to meet the needs of this population.

Further research that includes surveys to estimate the size of the transgender population is essential until such data is incorporated into the demographic census, as well as the development of more in-depth studies on self-inflicted and sexual violence, which have increased in this population.

Despite the limitations identified, it is expected that the study can raise awareness of the characteristics of the notifications of violence in the transgender population – which has evolved over the eight years following the inclusion of this population in the notification form – and thus contribute to supporting public policies aimed at addressing this issue.
