# Supplementary material for: Temporal trend and characteristics of notifications of self-inflicted and interpersonal violence in the transgender population in Brazilian municipalities, 2015-2022
Source: Epidemiol Serv Saude. 2024 Dec 6;33(spe1):e2024296. doi: 10.1590/S2237-96222024v33e2024296.especial.en (PMC11654394; doi:10.1590/S2237-96222024v33e2024296.especial.en)
Supplement: Translation

# Translation: Tendência temporal e características das notificações de violências autoprovocada e interpessoal na população transgênera nos municípios brasileiros, 2015-2022

**DOI:** 10.1590/S2237-96222024v33e2024296.especial.pt

**Authors:** Kelly Roberta Estrela Marinho, Jeane Tomazelli, Vania Reis Girianelli

**Affiliations:** 1 Fundação Oswaldo Cruz, Programa de Pós-graduação em Saúde Pública, Rio de Janeiro, RJ, Brasil; 2 Instituto Nacional de Câncer, Divisão de Ensino Stricto Sensu, Rio de Janeiro, RJ, Brasil; 3 Fundação Oswaldo Cruz, Departamento de Direitos Humanos, Saúde e Diversidade Cultural, Rio de Janeiro, RJ, Brasil

**Keywords:** Pessoas Transgênero, Violência de Gênero, Sistema de Informação em Saúde, Direitos Humanos, Epidemiologia Descritiva

## Abstract

**Objetivo:**

Analisar a tendência temporal e características das notificações de violências ocorridas na população transgênera no período de 2015 a 2022, nos municípios brasileiros.

**Métodos:**

Estudo epidemiológico do tipo painéis repetidos, com base em dados de violência ocorrida na população transgênera de 20 a 59 anos, disponíveis no Sistema de Informação de Agravos de Notificação. Realizada análise de tendência temporal anual por regressão linear generalizada, utilizando-se o método de Prais-Winsten e distribuição espacial dos municípios brasileiros com notificação.

**Resultados:**

As notificações de violência na população transgênera decresceram no período (1,7%; β = -0,07; p = 0,010), mas com aumento dos municípios notificantes (45,8%), da violência autoprovocada (28,9%; β = 2,21; p < 0,001) e sexual (β = 0,79; p < 0,001). A maioria dos agressores é do sexo masculino e com relacionamento afetivo, principalmente para mulheres transgêneras (43,4%; p < 0,001).

**Conclusão:**

A notificação da violência ainda não reflete a realidade dessa população, mas é o primeiro passo para a sua visibilidade e enfrentamento.

## Contribuições do estudo

**Principais resultados:**

Identificou-se que 1,7% das notificações de violência no país, ocorreram contra a população trans. A violência autoprovocada e a sexual aumentaram de 2015 a 2022. Quase metade dos municípios brasileiros já estão notificando violências que atingem essa população.

**Implicações para os serviços:**

A notificação contínua e com qualidade contribuirá para monitorar e conhecer a violência nesse grupo populacional, possibilitando direcionar os serviços à demanda específica dessa população.

**Perspectivas:**

O desenvolvimento de pesquisas sobre a população transgênera possibilitará conhecer e orientar ações especificas de saúde nesse grupo. As informações sobre violências nessa população contribuem para subsidiar as políticas públicas.

## INTRODUÇÃO

A violência é um conceito complexo cujos danos à saúde física e psicológica impactam, além da saúde e segurança, outras áreas da sociedade, como educação e economia, e por isso tem que ser uma das prioridades para as políticas públicas desenvolvidas no país.^1^ Desde 1893, o óbito por violência faz parte da lista de Classificação Internacional de Doenças.^2^ Apenas na década de 1990, a Organização Pan-Americana da Saúde, diante do aumento da morbimortalidade por violência na América Latina, passou a recomendar a inclusão da violência na agenda de intervenção^3^. Posteriormente, a Organização Mundial da Saúde considerou a violência como um problema de saúde pública que pode ser evitado, tendo organizado o primeiro relatório mundial contendo recomendações para o seu enfrentamento.^1^

A violência caracteriza-se pelo uso intencional da força ou poder que possa ocasionar lesão, morte, privação, deficiência ou dano psicológico.^1^ A violência autoprovocada é um ato de autoagressão do indivíduo, causando ferimentos leves ou graves e até mesmo comportamentos suicidas, enquanto na violência interpessoal há uso de força física, poder ou influência psicológica para dominação ou exclusão do outro. A violência interpessoal é subdividida em doméstica e comunitária. Na violência doméstica, os agressores são familiares, parceiros íntimos ou pessoas que convivam no espaço doméstico, mesmo que esporadicamente, como empregados e agregados, podendo esse tipo de violência ocorrer fora do ambiente familiar. Por seu turno, na violência interpessoal comunitária, os agressores são desconhecidos ou conhecidos, mas sem vínculo familiar ou afetivo.^4^

Em 2001, o Brasil instituiu a Política Nacional de Redução da Morbimortalidade por Acidentes e Violências,^5^ e em 2004 implantou a Rede Nacional de Prevenção da Violência e Promoção da Saúde.^6^ A partir de 2011, a notificação de violência autoprovocada e interpessoal integrou a lista de notificação compulsória^7^ e foi incluída no Sistema de Informação de Agravos de Notificação (Sinan), passando a conter registros de todo o país, embora a legislação já incorporasse a notificação de violência contra crianças e adolescentes, idosos, mulheres e de suicídios. Em 2015, para contemplar a Política Nacional de Saúde Integral de Lésbicas, Gays, Bissexuais, Travestis e Transexuais,^8^ foram incluídas, na ficha de notificação, as variáveis “orientação sexual” e “identidade de gênero”.^4^

A identidade de gênero é a forma como o indivíduo se percebe e se expressa socialmente, e quando ela difere do sexo biológico o indivíduo é denominado transgênero.^9^ Estima-se que 0,7% da população adulta brasileira é transgênera.^10^ Essa população é uma das mais vulneráveis à violência, devido à grande discriminação individual e institucional que vivencia.^11^ O Brasil é o país que mais mata pessoas transgêneras, principalmente travestis e mulheres transexuais, sendo marcadamente intolerante com a diversidade.^12,13^

A notificação de violência interpessoal e autoprovocada na população transgênera permite monitorar esse problema de saúde pública e pautar a gestão quanto às ações de intervenção necessárias para reduzi-la e alcançar sua prevenção.^14^ Este estudo tem por objetivo analisar a tendência temporal e características das notificações de violências ocorridas na população transgênera no período de 2015 a 2022, nos municípios brasileiros.

## MÉTODO

Estudo epidemiológico do tipo painéis repetidos, estudo híbrido que combina estudo transversal e de seguimento,^15^ sobre a violência autoprovocada e interpessoal na população transgênera de 20 a 59 anos, com registro no Sinan, no período de 2015 a 2022, com dados preliminares para 2021 e 2022. Os arquivos referentes aos microdados anonimizados compactados foram baixados do sítio eletrônico do Departamento de Informática do Sistema Único de Saúde, na primeira quinzena de dezembro de 2023. 

A tendência temporal anual para o período foi descrita tendo como variável independente o ano da notificação, e como variável dependente, a proporção de violências contra indivíduos transgênera em relação ao total de notificação da faixa etária estudada, bem como a proporção do tipo de violência (autoprovocada, interpessoal e ignorada) em trangênera e a proporção de cada natureza da violência interpessoal (física, psicológica etc.). A avaliação da tendência temporal foi realizada por regressão linear generalizada, utilizando-se o método de Prais-Winsten. No caso de tendência com significância estatística (p ≤ 0,05), utilizou-se a estatística de Durbin-Watson (d), considerando-se uma série de oito anos, em que resultados entre 1,332 e 2,668 confirmam que não há autocorrelação residual.^16^

A distribuição espacial dos municípios brasileiros com e sem notificação de violências (autoprovocada e interpessoal) contra a população transgênera de 20 a 59 anos foi apresentada em mapa coroplético, utilizando-se o programa TabWin versão 4.1.5, para o ano de início da série estudada (2015), antes da pandemia (2019), na pandemia (2020) e no final (2022). Também foi calculada a proporção de municípios de cada estado e região com notificação.

Adicionalmente, foram analisadas as variáveis disponíveis: gênero, características socioeconômicas (faixa etária, raça/cor da pele e escolaridade), mesmo município de residência e notificação (sim; não), características do agressor (quantidade, sexo e vínculo) e motivação para a agressão. Foi calculado o percentual de cada categoria das variáveis, estratificando-se as características socioeconômicas e demográficas por tipo de notificação (autoprovocada e interpessoal) e as características da violência interpessoal por gênero (homem transgênero e mulher transgênera, incluindo travesti). O teste qui-quadrado de Pearson foi utilizado para avaliar a existência de diferença estatisticamente significativa entre os estratos (p ≤ 0,05), com correção de Yates, se necessário. 

Os dados foram analisados no programa estatístico R versão 4.2.1 utilizando-se os pacotes foreign, MASS, prais e read.dbc.

## RESULTADOS

Foram notificados 2.778.017 casos de violência no período, sendo 1.535.329 na faixa etária de 25 a 59 anos (55,3%). Desses, 26.258 (1,7%) foram referentes à população transgênera, apresentando tendência de queda no período (β = -0,07; p = 0,010; d = 1,71) e com maior percentual de notificação em 2016 (2,2%) (Figura 1). Entre as notificações na população transgênera, a maioria foi de violência interpessoal (66,6%), mas com decréscimo no período (β = -1,94; p < 0,001; d = 2,21). O percentual de notificação de violência autoprovocada (28,9%), entretanto, tem aumentado (β = 2,21; p < 0,001; d = 1,88), representando 35% das notificações em 2022. Já o percentual de classificação ignorada quanto ao tipo de violência manteve-se estável (4,5%; p = 0,157).

**Figura 1:** Distribuição percentual das notificações de violência na população transgênera, com idade entre 25 e 59 anos, e por tipo de notificação segundo ano, Brasil, 2015 a 2022

A Figura 2 apresenta os mapas dos municípios brasileiros com notificações de violências que acometeram a população transgênera no grupo etário estudado. Em 2015, 13,2% dos municípios notificaram, aumentando para 18,2% em 2019 (antes da pandemia), caindo para 16,4% em 2020 (durante a pandemia) e atingindo 19,0% em 2022. No período 2015 a 2022, 45,8% dos municípios notificaram violências contra a população transgênera. A região Sudeste apresentou o maior percentual de municípios com notificação (61,3%), sendo esta proporção maior no estado do Rio de Janeiro (70,7%). A região Nordeste apresentou o menor percentual de municípios notificantes (37,8%), mas com alto percentual no estado do Rio Grande do Norte (79,2%), e baixo nos estados de Sergipe (24,0%), Piauí (15,2%) e Paraíba (12,6%). Mais da metade dos municípios da região Norte notificaram, com alto percentual para o estado de Roraima (80,0%). 

**Figura 2:** Municípios com notificação de violências autoprovocada e interpessoal na população transgênera com idade entre 25 e 59 anos, Brasil, 2015, 2019 (antes da pandemia), 2020 (na pandemia) e 2022

A notificação de violência autoprovocada foi proporcionalmente maior nos homens transgênero (22,4%; p < 0,001), menores de 40 anos (78,6%; p < 0,001) e brancos (45,9%; p < 0,001) do que para a violência interpessoal (Tabela 1). A escolaridade teve alto percentual de informação ignorada, sendo proporcionalmente maior para a notificação de violência autoprovocada (31,9%; p < 0,001). Cerca de 90% das notificações foram realizadas no município de residência, tanto para a violência autoprovocada quanto para a interpessoal (p = 0,094).

**Tabela 1:** Distribuição percentual das características sociodemográficas da população transgênera, segundo tipo de violência, Brasil, 2015 a 2022

Características	Tipo de violência		p-valor
	Interpessoal			Autoprovocada	
	n	%		n	%
**Gênero**					
Homem transgênero	3.142	18,0	1.702	22,4	< 0,001
Mulher transgênera	14.342	82,0	5.881	77,6	
**Faixa etária**					
20 a 39	13.112	75,0	5.962	78,6	
40 a 59	4.372	25,0	1.621	21,4	< 0,001
**Raça/cor da pele**					
Branca	6.188	35,4	3.481	45,9	< 0,001
Negra	10.032	57,4	3.638	48,0	
Amarela	144	0,8	58	0,8	
Indígena	205	1,2	69	0,9	
Ignorada	915	5,2	337	4,4	
**Escolaridade**					
Analfabeto	162	0,9	57	0,8	< 0,001
Fundamental incompleto	3.975	22,7	1.394	18,4	
Fundamental completo	1.305	7,5	449	5,9	
Médio incompleto ou completo	5.658	32,4	2.592	34,2	
Superior incompleto ou completo	1.350	7,7	674	8,9	
Ignorada	5.034	28,8	2.417	31,9	
**Mesmo município de residência e de notificação**					
Sim	15.807	90,4	6.803	89,7	0,094
Não	1.677	9,6	780	10,3	
**Total**	17.484	100,0	7.583	100,0	-

Múltiplos tipos de violência interpessoal ocorreram em 32,5% das notificações. A maioria foi de violência física (87,5%), seguida de violência psicológica (35,3%), mas ambas decresceram no período (β = -0,10; p = 0,041; d = 1,87 e β = -1,06; p = 0,003; d = 1,64, respectivamente) (Figura 3). A tortura também apresentou queda (β = -0,25; p = 0,011; d = 1,91), com maior percentual de notificação em 2015 (6,5%) e menor em 2021 (4,4%). Em contrapartida, a violência sexual aumentou (β = 0,79; p < 0,001; d = 2,04), com 8,5% das notificações no período, atingindo 11,5% em 2021. A notificação de violência sexual no período foi proporcionalmente maior para os homens transgênero (9,2%; p < 0,001) (dados não mostrados em tabelas). 

**Figura 3:** Distribuição percentual da natureza da notificação de violência interpessoal na população transgênera de 20 a 59 anos, por ano, Brasil, 2015 a 2022

A maioria das notificações de violência interpessoal envolveu apenas um agressor (69,0%) e do sexo masculino (80,7%), sendo que mais de um agressor e envolvendo ambos os sexos dos agressores foram proporcionalmente maiores para homem transgênero (34,4%; p < 0,001 e 3,7%; p = 0,026, respectivamente) (Tabela 2). Em relação ao vínculo do agressor com o(a) agredido(a), 41,0% envolviam relacionamento afetivo – cônjuge, ex-cônjuge, namorado(a), ex-namorado(a) –, sendo proporcionalmente maior para mulher transgênera (43,4%; p < 0,001); em seguida, desconhecidos (20,6%), com maior proporção para homem transgênero (24,7%; p < 0,001). A violência perpetrada por conhecidos e familiares também foi proporcionalmente maior para homem transgênero (21,6% e 12,5%, respectivamente; p < 0,001). A maioria dos motivos da violência foi ignorada (32,7%), sendo proporcionalmente maior para mulher transgênera (33,5%). 

**Tabela 2:** Distribuição percentual das notificações de violência interpessoal na população transgênera, por características dos agressores e da motivação, Brasil, 2015 a 2022

**Características**	**Total**		**Homem transgênero**		**Mulher transgênera**		**p-value**
	**n**	**%**	**n**	**%**	**n**	**%**	
Quantidade de agressores							
Um	11.409	69,0	1.909	60,8	9.500	66,2	< 0,001
Dois ou mais	5.133	31,0	1.080	34,4	4.053	28,3	
Ignorado	942	5,4	153	4,9	789	5,5	
**Sexo do(s) agressor(es)**							
Masculino	13.347	80,7	2.417	76,9	10.930	76,2	0,026
Feminino	2.629	15,9	466	14,8	2.163	15,1	
Ambos	566	3,4	117	3,7	449	3,1	
Ignorado	942	5,4	140	4,5	802	5,6	
**Vínculos do agressor**							
Familiares^a^	1.821	10,4	393	12,5	1.428	10,0	< 0,001
Relacionamento afetivo^b^	7.174	41,0	945	30,1	6.229	43,4	
Cuidador	27	0,2	7	0,2	20	0,1	
Conhecido(s)	2.943	16,8	680	21,6	2.263	15,8	
Desconhecido(s)	3.595	20,6	777	24,7	2.818	19,6	
Institucional^c^	446	2,6	91	2,9	355	2,5	
Outros^d^	369	2,2	76	2,4	293	2,0	
Ignorado	1.327	7,5	389	12,4	938	6,5	
**Motivo da violência**							
Sexismo	2.008	11,5	246	7,8	1.762	12,3	< 0,001
Transfobia	1.578	9,0	426	13,6	1.152	8,0	
Conflito geracional	2.565	14,7	492	15,7	2.073	14,5	
Situação de rua	946	5,4	246	7,8	700	4,9	
Deficiência	128	0,7	17	0,5	111	0,8	
Outros	4.547	26,0	813	25,9	3.734	26,0	
Ignorado	5.712	32,7	902	28,7	4.810	33,5	
**Total**	17.484	100,0	3.142	100,0	14.342	100,0	-

a) Pai, mãe, padrasto, filho(a), irmão(ã); b) Cônjuge, ex-cônjuge, namorado(a), ex-namorado(a); c) Patrão/chefe, relação institucional, policial/agente da lei; d) Inclui racismo, intolerância religiosa e xenofobia, sendo 0,1% de cada.

## DISCUSSÃO

O estudo identificou que 1,7% das notificações de violência autoprovocada e interpessoal foi registrada entre a população transgênera, atingindo 2,2% em 2016. Pesquisa realizada entre novembro e dezembro de 2018 estimou em 0,7% a população transgênera maior de 18 anos no país,^10^ com menor proporção para a região Centro-Oeste (0,2%). Destaca-se, contudo, que a população transgênera é uma das mais vulneráveis à intolerância e discriminação; portanto, é esperada maior proporção de notificação de violência do que nos demais grupos. Adicionalmente, a pesquisa^10^ foi realizada num período de ascensão da extrema direita no país, com discurso moral conservador e forte crítica à “ideologia de gênero”,^17^ o que pode ter inibido a resposta de alguns participantes. O percentual de municípios notificantes, entretanto, aumentou no período, atingindo 19,0% em 2022, mas quase metade dos municípios já notificaram no período. Ademais, cerca de 90% das notificações ocorreram no município de residência, sinalizando adesão aos serviços de saúde. Estudo realizado com notificação de violência interpessoal contra mulheres de 20 a 59 anos, no período de 2015 a 2021, identificou que 31,7% dos municípios brasileiros tinham registro para transgêneras.^18^

A proporção da notificação de violência autoprovocada tem aumentado, e foi proporcionalmente maior nos homens transgêneros, brancos e mais jovens do que para violência interpessoal. Resultados similares foram encontrados em estudo realizado com base em prontuários de ambulatório para transgênero no Distrito Federal,^19^ em que pensamentos suicidas foram mais frequentes nos homens transgêneros (80,5%), de 18 a 40 anos e com ensino médio incompleto ou superior (60,6%). Estudo realizado com adultos transgênero residentes nos estados de São Paulo e Rio Grande do Sul identificou que 67,7% apresentavam ideação suicida e 43,1% já haviam tentado o suicídio.^20^ Pesquisa realizada com mulheres transgêneras residentes em São Paulo constatou que o histórico de violência sexual foi um dos fatores associados à ideação e às tentativas de suicídio, mesmo após ajustado pelos demais fatores com significância estatística.^21^

A maioria das notificações de violência na população transgênera foi referente às mulheres, sendo 82,0% das violências interpessoais e 77,6% das autoprovocadas. Estudo realizado entre 2016 e 2020, utilizando-se dados do TabNet de violência interpessoal da cidade de São Paulo, também identificou maior percentual de notificação entre as mulheres transgêneras.^22^

A proporção de notificação de violência interpessoal na população transgênera decresceu no período, mas com aumento para violência sexual, sendo maior para os homens. A notificação de violência sexual, tem sido maior para mulheres cisgêneras do que transgêneras.^18^ O estupro corretivo, cuja intenção é mudar a sexualidade de forma a impor a heteronormatividade,^23^ é uma modalidade que tem sido noticiada para mulheres cisgêneras lésbicas, mas os homens transgêneros também tendem a estar sujeitos a esse tipo de violência. O estupro corretivo e coletivo já tem pena agravada no Código Penal.^24^

A violência física ainda é a mais notificada, mas apresentou uma queda, de 2019 a 2021, que pode estar relacionada à dificuldade de acesso aos serviços de saúde devido à epidemia de covid-19. A equiparação de atitudes ofensivas, preconceituosas e de violência contra pessoas LGBTQIAPN+ ao crime de racismo, em 2019,^25^ também pode ter influenciado a redução das notificações. A queda nas notificações não significa necessariamente reduções de práticas violentas na população transgênera, cuja retomada da violência física em 2022 já se aproxima ao início da série (2015). Estudo que analisou a violência contra as mulheres constatou maior proporção de notificação de violência física contra as mulheres transgêneras.^18^

A violência psicológica foi a segunda mais frequente, expressando o seu reconhecimento tanto pela vítima quanto pelos profissionais, mas, diferentemente da violência física, manteve-se em queda em 2022. A violência psicológica costuma aparecer no início do ciclo de agressão, principalmente nos relacionamentos afetivos, e, portanto, tende a ser mais invisibilizada. Ela costuma ser mais frequentemente revelada em estudos com entrevistas,^26^ e muitas vezes pode ser identificada apenas pelo(a) entrevistador(a). 

A maioria dos agressores foi do sexo biológico masculino (80,7%) e com relacionamento afetivo com a vítima (41,0%), sendo proporcionalmente maior para mulheres transgêneras (p < 0,001). Estudo restrito à violência contra mulheres constatou que a agressão oriunda de pessoas com as quais a vítima tinha vínculo afetivo foi maior para as transgêneras.^18^ Uma revisão sistemática com metanálise sobre violência pelo parceiro íntimo na população transgênera^27^ encontrou que a prevalência média por esse tipo de agressor, ao longo da vida, foi de 37,5%, e que a população trans tem uma probabilidade maior de sofrer violência por parceiro íntimo em comparação à população cisgênera. Esse estudo incluiu 74 artigos de diversos países publicados até 2019, sendo dois brasileiros, mas o enfoque principal era o HIV. 

O motivo da violência não foi registrado em 32,7% das notificações, inviabilizando o conhecimento das principais causas da agressão. A notificação da violência perpetrada em razão da identidade de gênero (transfobia), às vezes presumida pelo agressor em relação à vítima, é fundamental para subsidiar as políticas públicas para o enfrentamento dessa discriminação. A escolaridade também apresentou alto percentual de registros ignorados, o que tem sido observado em estudos baseados em sistemas de informação. 

O baixo preenchimento de alguns campos da ficha de notificação é uma das limitações do estudo, bem como o provável erro de preenchimento, pois no tema há conceitos que podem ainda não ser de domínio dos profissionais de saúde, como tem sido evidenciado em alguns estudos.^28,29^ A ausência de informação sobre a identidade de gênero no censo demográfico também inviabiliza estimar a prevalência de violência para a população transgênera, informação fundamental para subsidiar as políticas públicas. As estimativas existentes, apesar dos esforços de representatividade, incluem um pequeno tamanho de amostra e tendem a se concentrar nos grandes centros urbanos. 

Adicionalmente, a provável subnotificação deve ser maior nesse tipo de agravo, pois depende do reconhecimento da vítima e do profissional de saúde, situação que nem sempre é percebida por ambos. A parceria com as organizações não governamentais, incluindo a notificação, como previsto na legislação, poderá contribuir para minimizar as subnotificações existentes, melhorando a qualidade das informações e o direcionamento dos serviços à demanda dessa população.

Pesquisas futuras que contemplem a realização de inquéritos para estimar o tamanho da população transgênera são fundamentais, enquanto os dados a respeito não são incorporados no censo demográfico, bem como o desenvolvimento de estudos mais aprofundados sobre as violências autoprovocada e sexual, que têm aumentado nessa população.

Apesar das limitações identificadas, espera-se que o estudo possa dar visibilidade às características da notificação de violência na população transgênera – que evoluiu nos oito anos após a inclusão dessa população na ficha de notificação –, e dessa forma contribuir para subsidiar as políticas públicas para enfrentamento desse agravo.
